# Non-lupus full-house nephropathy—immune dysregulation as a rare cause of pediatric nephrotic syndrome: Questions

**DOI:** 10.1007/s00467-021-05359-3

**Published:** 2021-12-17

**Authors:** Orsolya Horváth, George S. Reusz, Veronika Goda, Kata Kelen, István Balogh, Magdolna Kardos, Krisztián Kállay, Áron Cseh, Attila J. Szabó, Gergely Kriván

**Affiliations:** 1 1st Department of Pediatrics, Semmelweis University, 53-54 Bókay János Street, 1083 Budapest, Hungary; 2 Pediatric Hematology and Stem Cell Transplantation Unit, Central Hospital of Southern Pest, National Institute of Hematology and Infectious Diseases, Budapest, Hungary; 3 Department of Laboratory Medicine, Division of Clinical Genetics, Department of Human Genetics, Faculty of Medicine, University of Debrecen, Debrecen, Hungary; 4 2Nd Department of Pathology, Semmelweis University, Budapest, Hungary

**Keywords:** Nephrotic syndrome (NS), Lupus nephritis, “Full-house” nephropathy (FHN), Primary immunodeficiency (PID), Autoimmunity

## Case

**Fig. 1:**
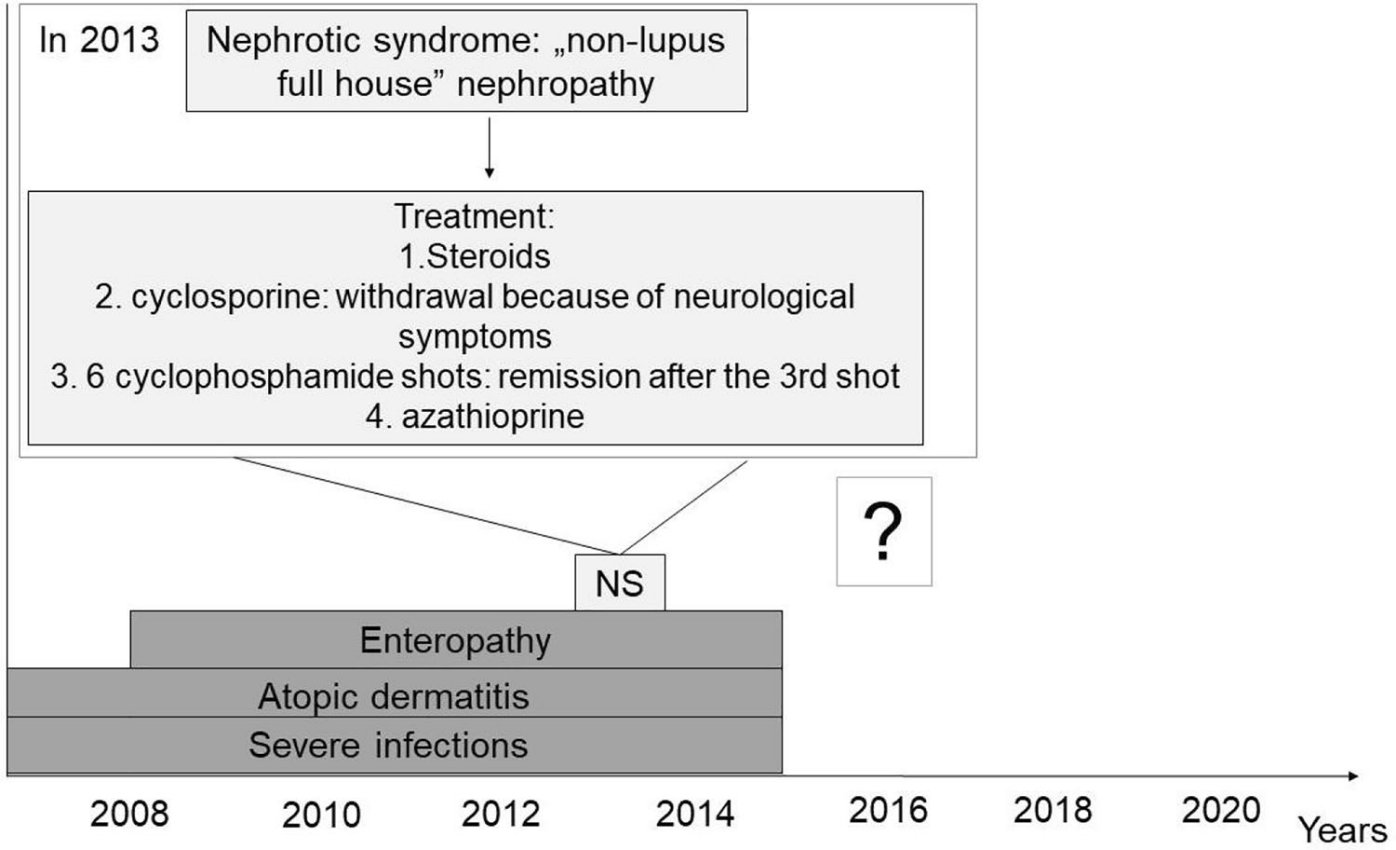
Patient flow chart. NS indicates nephrotic syndrome

**Fig. 2:**
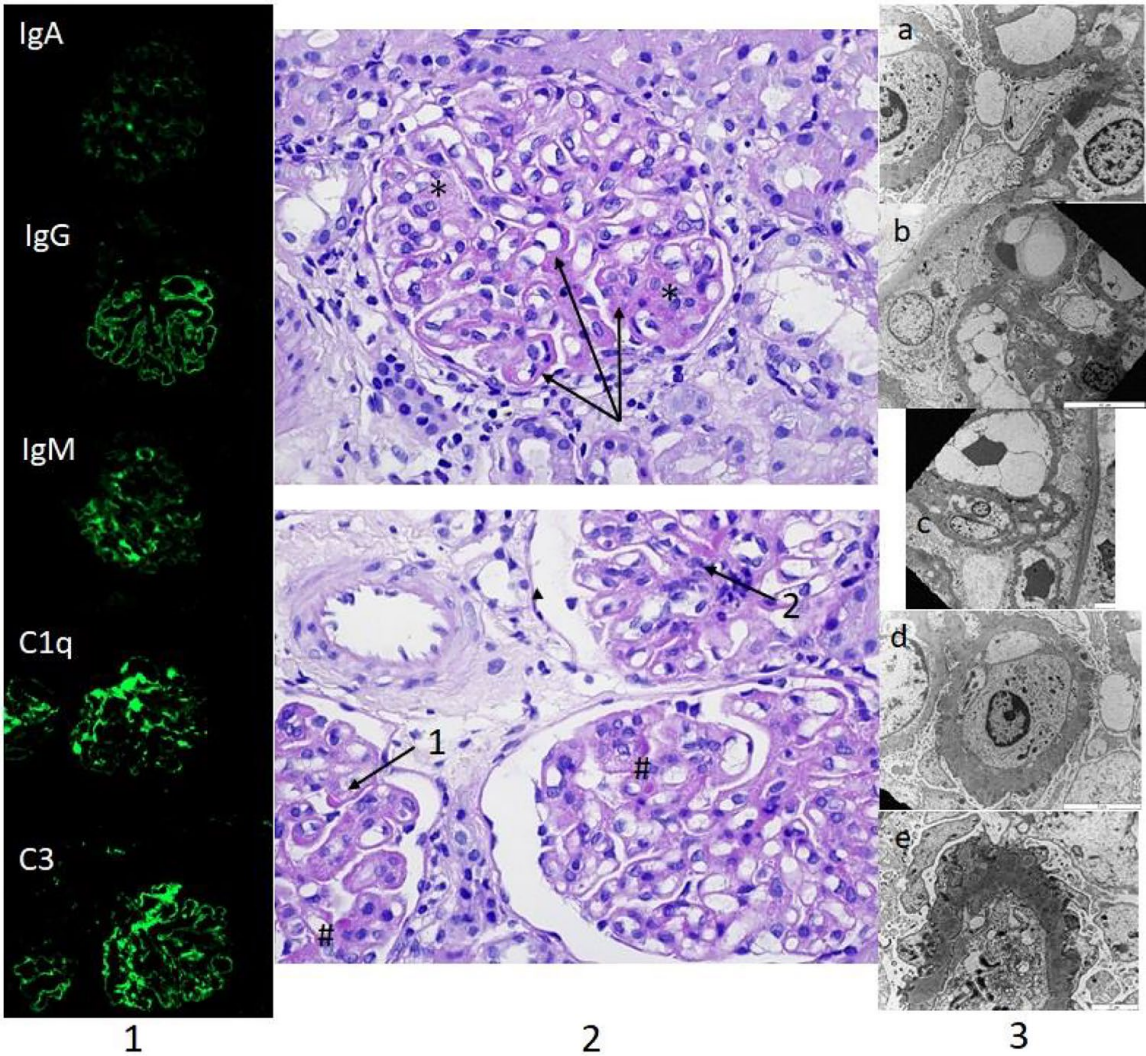
Histopathologic findings in the non-lupus “full-house” nephropathy case. (1) “Full-house staining” by immunofluorescence: deposits are seen mainly along the glomerular basement membrane and scattered at mesangial sites. (2) Light microscopy showing diffuse mesangial matrix expansion in the glomeruli (arrows, middle, upper panel) and focal endocapillary/mesangial proliferation (asterisks); diffuse thickening of the glomerular basement membrane (arrow 1) with hyaline pseudothrombi (number sign, arrow 2, middle, lower panel). PAS, 600 × . (3) Electron microscopy showing (a) deposits in subepithelial locations and one in subendothelial location with extensive podocyte effacement in the glomerular capillary loop, (b) deposits in mesangial locations, (c) focal mesangial interposition, and (d, e) subepithelial deposits in different stages, with extensive podocyte effacement

We present the case of a boy, who was first referred to our nephrology department in 2013 at the age of 6 with periorbital oedema, nephrotic range of proteinuria (100 mg/m^2^/h), glomerular haematuria, hypalbuminaemia and hyperlipidaemia. Clinical criteria of nephrotic syndrome (NS) with normal kidney function were fulfilled (Fig. 1). After an unsuccessful initial steroid treatment (2 mg/kg prednisone for 4 weeks), kidney biopsy was performed. Histology was evaluated by a clinical pathologist in our hospital according to the 2004 WHO classification [1]. “Full-house” nephropathy (FHN) was diagnosed by immunofluorescent staining with extensive complement (C1q, C3) and immunoglobulin deposition. IgA, IgG, IgM, C3 and C1q deposits were simultaneously present without clinical signs of systemic lupus erythematosus (SLE) and with negative autoantibody serology (ANA, anti-DNA antibodies, ANCA) (Fig. 2). Consequently, non-lupus FHN was diagnosed. It was classified as diffuse membranous glomerulonephritis (GN) with focal segmental endocapillary proliferative lesions [1]. Cyclosporine was added as adjunctive therapy [2]. However, cyclosporine had to be stopped after 3 weeks due to seizures, which was diagnosed as posterior reversible encephalopathy, also confirmed by MRI. Intravenous cyclophosphamide pulses were administered (a total of six pulses of 500 mg/m^2^) followed by azathioprine maintenance therapy. From the third month of treatment, proteinuria decreased gradually and NS went into remission, without deterioration of kidney function.

The patient had other important and variable symptoms as well. He had been hospitalized several times since infancy for severe enteropathy and atopic dermatitis (Fig. 1). No allergic or infectious causes of diarrhoea were found and in terms of nutrition, he only tolerated elementary formulas. The enteropathy worsened further from the age of 7, with concomitant failure to thrive. Endoscopy performed at the age of 8 (in 2015) revealed severe terminal ileitis and colitis, suggesting an autoimmune origin of diarrhoea. He was fed via a percutaneous endoscopic gastrostomy with elementary amino acid–based formula.

Furthermore, he had also been hospitalized since birth for serious infections, including fungal infections, *Candida albicans* sepsis and several episodes of viral gastroenteritis.

The lack of response to first-line treatment of nephrotic syndrome, insufficient weight gain with severe diarrhoea, and the variable and severe infections pointed to the possibility of primary immunodeficiency. Flow cytometry was performed first at the age of 9, showing T cell, CD4 and CD8 cell counts (1022, 503 and 403 cells/μL) and CD4/CD8 rate (1.25) within normal ranges. However, the percentage of regulatory T cells was at the lower limit of normal (CD4 + /CD25 + /CD127dim cells 4.68% (ref. range 4–9%)).

## Questions

1. What is the most likely diagnosis?
2. What is the possible underlying mechanism of kidney involvement?
3. What is the treatment and the prognosis of the disease?

## Data Availability

Not applicable
